# Noma as a complication of false teeth (Ebiino) extraction: a case report

**DOI:** 10.1186/s13256-017-1276-5

**Published:** 2017-04-17

**Authors:** Martin Tungotyo

**Affiliations:** Mbarara University of Science and Technology, Mbarara, Uganda

**Keywords:** Ebiino, False tooth extraction, Traditional practice, Case report, Noma

## Abstract

**Background:**

Ebiino, also known as false tooth extraction, is a traditional practice done mainly in the remote areas of African countries, including Uganda. It involves the extraction of tooth buds in babies with common childhood illnesses such as fever, cough, and diarrhea. It is thought that the tooth buds are responsible for the ailments seen in these infants. The practice is performed by traditional healers using unsterile instruments. The complications associated with this dangerous practice have been mentioned in the literature and include anemia and septicemia, among others. This case report describes a baby with noma, an orofacial gangrenous infection.

**Case presentation:**

A 16-month-old girl from western Uganda belonging to the Banyankole ethnic group was admitted to Mbarara University Teaching Hospital with a 5-day history of a dark lesion on the left cheek. The lesion had started from the left upper gum at the site where a tooth bud had been extracted 1 week prior to admission. The child had experienced occasional cough and fever and also had erupting tooth buds. These tooth buds had been seen as the cause of the cough and fever by the traditional herbalist; hence, they were extracted. An unsterile instrument had been used for the procedure. At the hospital, a local examination showed necrotic tissue involving the left cheek and extending into the left upper gingival area of the girl’s mouth. A clinical diagnosis of orofacial gangrene (noma) was then made.

**Conclusions:**

Ebiino, or false tooth extraction, is still practiced in some remote areas of Uganda. Noma has been mentioned as a possible complication of this traditional practice; however, case reports in the literature are scant. Public awareness of the dangers of this practice is therefore still required to prevent this dangerous complication.

## Background

“False teeth” (Ebiino) refers to gingival swelling that occurs during eruption of the primary canine teeth in infants and consists of extraction of deciduous canine tooth buds [1]. This practice, which is part of infant oral mutilation, is a relatively common practice in African countries with an incidence that varies from place to place, ranging between 15% and 80%, especially including Angola, Tanzania, Somalia, Kenya, Sudan, Nigeria, and Uganda [2]. It has also been reported in some non-African countries, including The Maldives, the United States, New Zealand, Israel, and Sweden, especially among the migrant population [2]. In Uganda, the practice was first reported among the Acholi people in Northern Uganda. However, the practice spread throughout the country and has been reported in places such as Mbarara in Western Uganda and Tororo in Eastern Uganda [1]. The practice arises from the belief that these “killer” canines cause fever, diarrhea, and any other infant illness, thus necessitating their removal, usually by traditional herbalists using unclean instruments and fingernails [1]. In Bushenyi district in Western Uganda, a study showed that more than one in two of the households had a child younger than 5 years old who had had false teeth in the last 5 years as of 2007, with more than 80% of the respondents using traditional medicine alone or in combination with modern medicine to treat “false teeth disease” [3]. This shows that the practice is still very popular in this population in Western Uganda.

The complications attributed to false teeth extraction are numerous and can be either local or systemic. They include anemia, pneumonia, meningitis, and septicemia, among others [4]. A study in a Northern Uganda hospital showed that complications from Ebiino or false teeth were the eighth most frequent cause of admission to the pediatric ward, with septicemia and anemia being the most frequent complications [1]. Noma (cancrum oris) is an orofacial gangrene that, during its fulminating course, causes progressive and mutilating destruction of the infected tissues [5]. This devastating disease, without appropriate treatment, has a mortality rate of 70 to 90%, and the survivors experience the twofold affliction of orofacial mutilation and functional impairment, which requires a time-consuming, financially prohibitive surgical reconstruction [6]. Noma has been mentioned as a possible complication of intraoral mutilation [2]. However, case reports on noma as a complication of Ebiino are scant. We describe a case of a patient with noma as a complication of Ebiino, a common traditional practice.

## Case presentation

A 16-month-old girl from Ankole in Western Uganda was admitted to the surgical ward of Mbarara University Teaching Hospital in Western Uganda with a dark lesion on the left cheek. This lesion had been present for 5 days prior to admission. It had started as a small red lesion on the left side of the upper gum and had quickly spread to the inner cheek. The child had been subjected to a tooth bud extraction on that side of the gums by a traditional herbalist 1 week prior to admission. The procedure had been done to cure Ebiino, a diagnosis that was reached by the traditional herbalist after the parents reported that the child was experiencing cough and flu and that they had spotted erupting tooth buds.

Associated with this dark lesion was a high-grade intermittent fever and pain. The girl’s feeding was also described as difficult, owing to her pain, but manageable. The child is the first born of the family and was up-to-date with her growth milestones and immunizations. Her mother has never attended any formal education and is a small-scale subsistence farmer together with the father of the child. The family hails from a village in Bushenyi district, which is located in Western Uganda.

A physical examination revealed that the child was in fair general condition. She had features of malnutrition that included brown, sparse hair, and she weighed 7.3 kg, which is below the fifth percentile on the weight-for-age chart adopted from the National Centre for Health Statistics. This showed failure to thrive. The child also had moderate pallor of the mucous membranes and was afebrile with a temperature of 36.6 °C.

Locally, she had a dark necrotic patch on the left cheek that involved most of the upper lip and nose and extended into the left upper gingival area. The lesion was generally oval in shape and measured about 8 cm × 5 cm. It was tender to touch. The rest of the local examination was unremarkable.

**Fig. 1:**
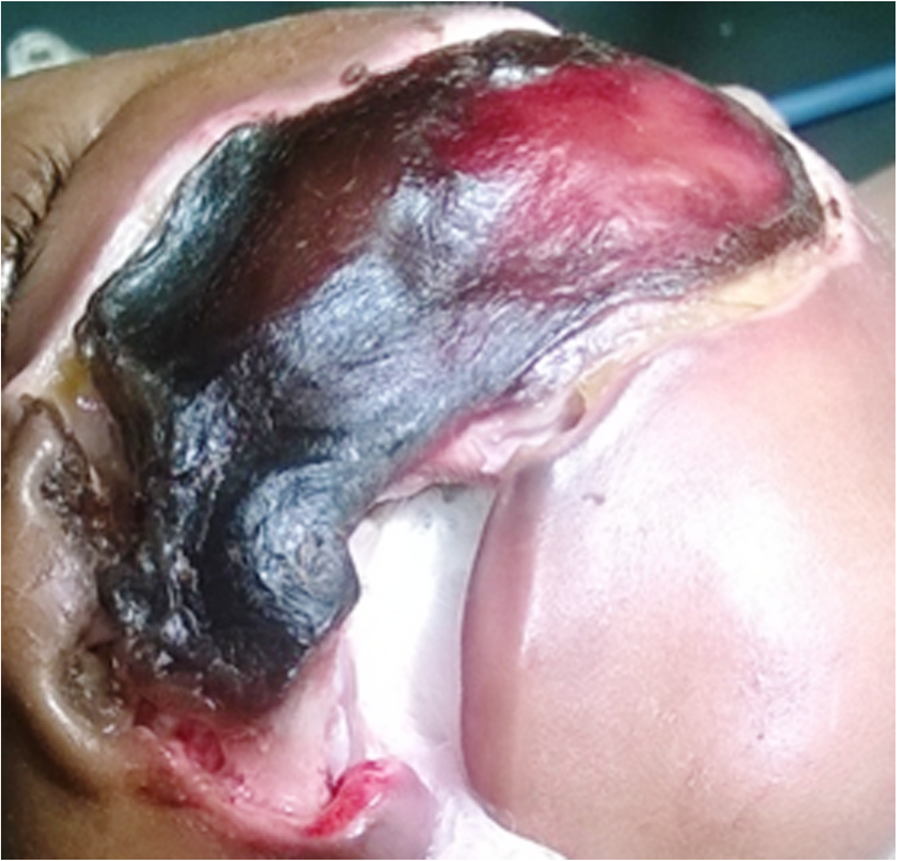
Preoperative photograph showing necrotic tissue on the left cheek

**Fig. 2:**
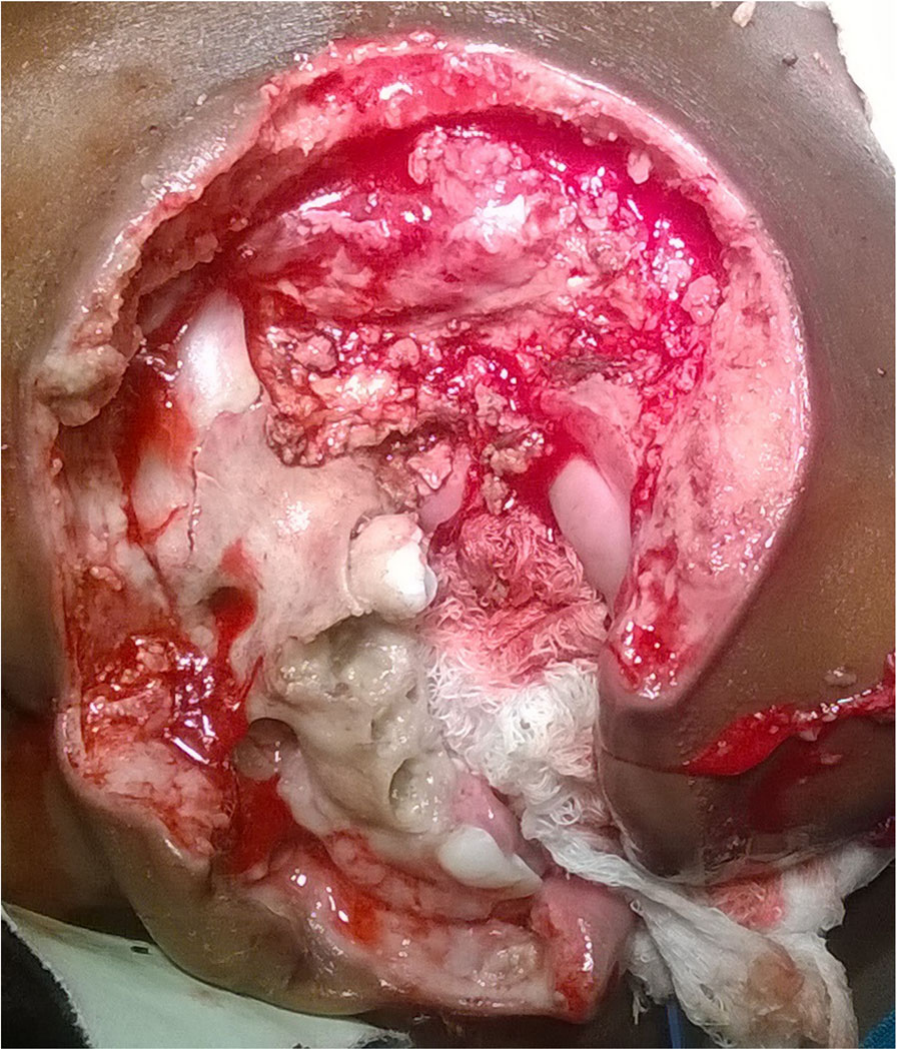
Photograph taken after debridement showing the extent of tissue destruction

The history and findings pointed to the diagnosis of noma. The child was admitted to our hospital and started on intravenous ceftriaxone 400 mg once daily as well as rectal paracetamol for pain. A blood sample was also taken for a complete blood count, which showed mild leukocytosis and an estimated hemoglobin level of 10 g/dl. Radiology services were not available at the hospital at the time, and the parents could not afford to have it done outside the hospital. Surgical debridement was also done (Fig. 1). The necrotic tissue was removed to show the full extent of involvement (Fig. 2). A nasogastric tube was inserted to aid feeding. The child was then referred to a specialist hospital for more rehabilitation and reconstructive surgery.

## Discussion

False teeth (Ebiino) refers to gingival swelling that occurs during eruption of the primary canine teeth in infants and consists of extraction of deciduous canine tooth buds [1]. This fairly common practice has been recorded mainly in African countries, including Ethiopia, Malawi, Uganda, South Sudan, and Kenya, among others [2]. Reports in the literature describe that the reason why this practice is done is the belief that these false teeth are responsible for a number of infant illnesses, including fever, diarrhea, and malnutrition, among others, hence necessitating their removal, usually by traditional herbalists who use unsterile instruments that include bicycle spokes, knitting needles, scissors, broken glass, or fingernails [2, 7].

In Uganda, the regions in which this practice has been described include Northern, Eastern, and Western Uganda [1]. In some areas, the frequency of Ebiino is one in every three children [3]. In a study done in Bushenyi in Western Uganda, where our patient comes from, more than one in two households has a child younger than 5 years of age who had had false teeth. The same study authors stated that more than 80% of the respondents sought either a traditional herbalist alone or in combination with a modern medical worker. In Uganda, traditional healers are often the first point of contact for those seeking health care provision because they share the same beliefs, culture, and values [3].

Low level of education was found to favor the occurrence of these false teeth [8]. The lack of education favoring the occurrence of the false teeth is apparent in the case of our patient because the parents had not received any formal education.

The complications arising from this practice have been thought to be due to the use of unsterile instruments. Some of the complications described include septicemia, anemia, osteomyelitis of the maxilla and mandible, tetanus, and hemorrhage [2, 7]. A study in Northern Uganda showed that 14.5% of infants with complications arising from traditional practices including treatment of false teeth died [1]. Our patient developed noma as a complication of Ebiino.

Noma (cancrum oris) is a devastating gangrenous disease that leads to severe destruction of tissue in the face and is associated with high morbidity and mortality [9]. The disease is most common in sub-Saharan Africa and has been described as a scourge in communities with poor environmental sanitation [10]. The incidence is largely unknown, but literature reports have quoted a yearly incidence of 140,000 cases with a mortality of 90% [5]. An estimated 770,000 persons are surviving with sequelae [11]. The cause is unknown, but a combination of several elements of plausible etiology have been identified, including malnutrition, which our patient experienced, as well as a compromised immune system, poor oral hygiene, and lesions of the gingival-mucosal barrier [9].

Noma begins with ulceration of the gingivae in a wide variety of forms. If the condition is detected at the gingival stage, progression can be prevented by local disinfection, common antibiotics, and immediate nutritional rehabilitation [9]. Acute necrotizing gingivitis and oral herpetic ulcers are considered the antecedent lesions, and ongoing studies suggest that the rapid progression of these precursor lesions to noma require infection by a combination of microorganisms, with *Fusobacterium necrophorum* and *Prevotella intermedia* as the suspected key players [10].

If left untreated, the gingival condition can progress to noma proper through transmission of the soft tissues in contact with gingival lesions [12]. In this very painful stage, the cheeks or lips begin to swell, and the patient’s general condition deteriorates. Within a few days, the swelling greatly increases, and a blackish furrow appears where loss of substance is occurring. The gangrenous process sets in and leaves a gaping hole in the face after the scab falls off [12].

In the acute stage, the role of surgery is a minor one, with wound care and very occasionally treatment of hemorrhage [13]. For those who survive and develop a mutilated and disabled face, reconstructive surgery may improve their fate significantly [13].

The only truly effective approach to the problem of noma throughout the world is prevention [5]. Noma can be prevented through promotion of national awareness of the disease; poverty reduction; and improved nutrition, promotion of exclusive breastfeeding in the first 3–6 months of life, optimum prenatal care, and timely immunization against childhood diseases [10].

In our patient, addressing the traditional practice of Ebiino would have gone a long way in preventing the development of noma as a complication of the practice. A study done in a Western Ugandan community, where our patient is from, led to a number of conclusions regarding Ebiino, including the following, among others: that the concept and beliefs of false teeth (Ebiino) were still widespread in that community; that children younger than 2 years of age were still more vulnerable to those beliefs; that there was still a strong association of false teeth as a cause of diarrhea, acute respiratory infection, fever, loss of appetite, and malnutrition in children in that community; that traditional pediatric oral surgery was still widespread in that community; and that community knowledge on preventive methods of false teeth was still scanty [8].

## Conclusions

We have described a case of a patient with noma as a possible deadly complication of the traditional practice regarding false teeth (Ebiino) that is still prevalent in some rural communities in Uganda. More therefore needs to be done to address this common practice of false teeth extraction to prevent this terrible complication of noma.
